# Effects of Graded Exergames on Fitness Performance in Elementary School Children With Developmental Coordination Disorder

**DOI:** 10.3389/fspor.2021.653851

**Published:** 2021-04-22

**Authors:** Bouwien Smits-Engelsman, Emmanuel Bonney, Gillian Ferguson

**Affiliations:** ^1^Department of Health and Rehabilitation Sciences, Faculty of Health Sciences, University of Cape Town, Cape Town, South Africa; ^2^Elison Laboratory for Developmental Brain and Behavior Research, Institute of Child Development, University of Minnesota, Minneapolis, MN, United States

**Keywords:** fitness performance, children, active video game, developmental coordination disorder, training intensity, balance, agility

## Abstract

Developmental Coordination Disorder (DCD) is a common childhood disorder affecting movement and coordination skills, fitness, and academic performance. Increased physical fitness may have a positive influence on physical and mental health outcomes in children with DCD. Yet, little has been done to develop interventions to improve fitness performance in this group. The purpose of this study was to determine the effects of graded exergames in 7 to 12-year-old children with DCD and typically developing (TD) peers. Participants (32 DCD and 28 TD children) received a 30-min training session twice weekly for 10 weeks. Performance on motor coordination (MABC-2 test), balance, aerobic, and anaerobic fitness tests were assessed at the beginning and end of training. In addition, enjoyment and perception of exertion were measured for each participant during the training period. Both children with DCD and TD children significantly improved on motor coordination, balance, aerobic, and anaerobic fitness at the end of the training. A significant Group by Time interaction was observed on the MABC-2 total [*F*_(1, 55)_ = 13.19; *p* < 0.001] and balance scores [*F*_(1, 55)_ = 26.83; *p* < 0.0001], with the DCD group demonstrating larger improvements than the TD children. Both groups enjoyed the program throughout the training period even though they rated the training to be of high intensity. These findings indicate that graded exergames may serve as potential treatment for impaired fitness in children with DCD. Regular participation in graded exergames in school settings may be needed to enhance and maintain fitness performance in young children with and without DCD.

## Introduction

Children with developmental coordination disorder (DCD) exhibit severe motor clumsiness that interferes with academic achievement and the activities of daily living (American Psychiatric Association, 2013). DCD occurs in ~5–6% of children worldwide and is not explained by medical conditions or low IQ (American Psychiatric Association, 2013). Poor fitness performance is well-documented in children with DCD in both high- and low-income countries (Faught et al., 2005; Tsiotra et al., 2009; Rivilis et al., 2011; Ferguson et al., 2014; Lifshitz et al., 2014; Farhat et al., 2015). In general, children with DCD are more likely to have decreased physical fitness compared to peers with typical development (TD), perhaps due to an activity deficit resulting from poor motor proficiency and withdrawal from physical activity (Hay and Missiuna, 1998; Tsiotra et al., 2009; Cairney et al., 2017). Despite the enormous number of interventions developed to address DCD symptomatology, little research has been done to address DCD-related fitness impairments. Searching for effective methods to increase fitness performance is critical if we are to improve health and wellness in children with DCD.

Physical fitness consists of a set of measurable characteristics that people gain through various physical efforts (Deuster, 1997; Corbin et al., 2000). These include components such as cardiovascular endurance, body composition, muscle strength, endurance, flexibility, balance, coordination, agility, and reaction time (Deuster, 1997; Corbin et al., 2000). Among children, higher levels of physical fitness have been associated with healthy body weight, optimal psychological and bone health, and lower risk for obesity and cardiovascular diseases (Boreham et al., 2001; Biddle et al., 2004; Ortega et al., 2008; Janssen and LeBlanc, 2010). Additionally, adequate physical fitness is positively associated with high academic achievement (Castelli et al., 2007; Wittberg et al., 2012). Despite these benefits, recent studies have shown a decline in physical fitness among children worldwide (Lang et al., 2018; Tomkinson et al., 2019). It is therefore necessary to maximize efforts that will ensure that children of all abilities and socioeconomic backgrounds are provided with an opportunity to increase physical fitness and to participate in meaningful daily activities (Faigenbaum et al., 2011).

Exercise is the most frequently used treatment for children with DCD. Previous exercises that have been tested in individuals with DCD vary in type, intensity, frequency, and duration (Smits-Engelsman et al., 2018). Two very different popular approaches to exercise in the DCD literature are task-oriented functional exercises and active video games or exergames (Blank et al., 2019). While task-oriented exercises have been reported to produce greater improvements in motor coordination compared to exergames, evidence shows that exergames can serve as a useful adjunct to therapy (Blank et al., 2019). Unfortunately, there is currently little or no evidence to guide caregivers (health professionals, educators, coaches/physical trainers) to select the most effective type of exercise for the treatment of impaired physical fitness in young children with DCD. This lack of evidence suggests that treatment for poor fitness performance may be sub-optimal.

Active video games have been proven to be safe, engaging, enjoyable, and beneficial for improving motor coordination in children and adolescents with DCD (Ferguson et al., 2013; Hammond et al., 2014; Bonney et al., 2017a; Mentiplay et al., 2019). More recently, graded active video exercises have been demonstrated as a feasible approach to promoting physical fitness in youth with DCD (Bonney et al., 2018). However, we are not aware of any published research that has tested graded active video exercises in elementary school-aged children with or without DCD. Therefore, the purpose of this study was to determine the effects of graded active video exercises on health- and skill-related fitness measures in children with DCD and typically developing peers. Specifically, the following objectives were pursued: (1) to determine whether elementary school-aged children would tolerate graded active video games, (2) to quantify exercise intensity during the training period, and (3) to examine changes in physical fitness and motor performance at pre- and post-intervention.

## Materials and Methods

### Study Design

This study was conducted as a pre-post design.

### Participants

Thirty-two (*n* = 32) elementary school children (aged 7–12 years) with DCD (15 boys, 17 girls) and twenty-eight (*n* = 28) age- and gender-matched TD children (13 boys, 15 girls) attending school in an economically-deprived area of Cape Town, South Africa, participated in this study. The study was approved by the Human Research Ethics Committee (HREC) of the University of Cape Town, South Africa (HREC: 209/2018). Parents provided written consent, and each child gave assent before participation. Sample size was established with G^*^Power 3.1. (Faul et al., 2007) based on the assumption of an effect size of 0.7 and at least 90% power. Thus, a sample size of 24 children per group was deemed adequate to examine the hypothesis. All 32 children meeting the DCD criteria were offered the intervention.

### Procedure

#### Identification of Participants

**Figure 1:**
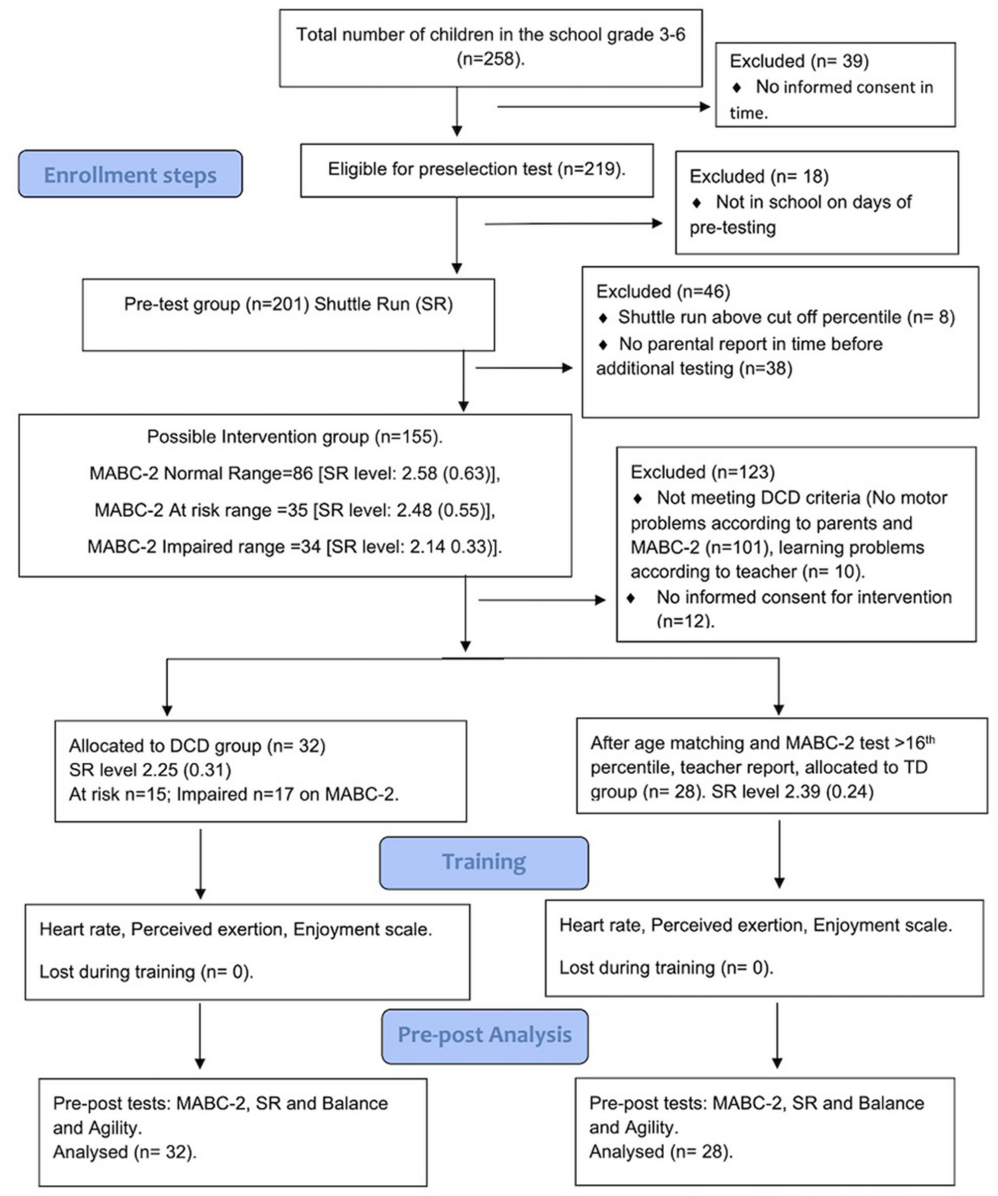
Consort statement depicting the enrollment steps.

Participants were identified using a three-step process (see Figure 1). First, children were tested on the 20 m shuttle run (SR) test after consent procedures had been completed. Second, children who performed below the 20th percentile on the SR test (fitness performance was evaluated based on VO_2_ max percentile scores published by Kolimechkov et al., 2019) and whose parents had completed a demographic questionnaire were assessed on the Movement Assessment Battery for Children, second edition (MABC-2 test) (Hendersen et al., 2007). The parental demographic questionnaire (Ferguson et al., 2013; Bonney et al., 2017b) asked about the pregnancy history of the child's mother, presence of visual, auditory, intellectual, or motor difficulties, established medical diagnoses (e.g., cerebral palsy), and whether the child experienced movement difficulties. Last, children whose parents gave informed consent to undergo additional testing were further evaluated for DCD using the DSM-5 diagnostic criteria (American Psychiatric Association, 2013) and were asked to take part in the training. Briefly, children with movement difficulties reported by their parents and who scored below the 16th percentile on the MABC-2 test; whose parents reported no diagnosis of a significant medical condition known to affect motor performance, visual, auditory, or attentional problems; and whose teacher confirmed the absence of cognitive impairment were identified as having DCD. The children with DCD were then matched with typically developing (TD) peers whose motor performance was ≥25th percentile on the MABC-2 test. The TD children had no problems with academic work (as confirmed by their class teacher), and their parents reported no diagnosis of a significant medical condition known to affect motor performance. For ethical reasons, we invited all children who met the DSM-5 diagnostic criteria for DCD to participate in the study. However, children were excluded if they had any documented medical condition that hindered their participation in the training. Selected children were also assessed on the balance and agility tests described below.

#### Assessments

All pre- and post-tests were administered by a team of trained physical therapists not involved in the training. Testing was spread over several days (14 days) to avoid fatigue. The MABC-2 and Foam Balance tests were administered individually. Sprints and agility tests were performed in pairs at the school's playground. For these tests, children were tested in alternating. Thus, while one child was being tested, the other was allowed to rest. The 20 m shuttle run test was done in groups of four or five children supervised by three or four testers.

Post-tests were planned to take place in the last 3 weeks before the school holidays. However, several unanticipated events occurred in this period, including water shortages, poor sanitation, interruption of electricity supply, and unanticipated community protest action regarding poor municipal service delivery resulting in the temporary closure of the school over safety concerns. Consequently, post-testing was stopped after the first week of testing. Furthermore, no testing was allowed after the children returned to school to complete the last quarter of the academic year. This is because research activities are usually not permitted in schools at this time of the year in the Western Cape Province of South Africa. In view of this, many of the children had incomplete data. We did not exclude children with incomplete test results because at least one post-test result was available for each child (see Table 1 for details of the study design).

**Table 1:** Study design.

**Activity**	**Description**	**Duration**
Pre-selection assessments	Children were assessed on SR and MABC-2 tests	2 weeks
Pre-training assessments	Children were assessed on balance and agility performance	2 weeks
Training	Week 1: Gaming with no extra challenge	
	Week 2: Gaming with no extra challenge	
	Week 3: Gaming on Airex Mat (L 50 × W 40 × H 1.5 cm)	
	Week 4: Gaming on Airex Mat (L 50 × W 40 × H 1.5 cm)	
Holidays	Students went on holidays; training was halted during this period	2 weeks
	Week 5: Gaming on Foam pad (L 47 × W 39 × H 6 cm)	
	Week 6: Gaming on Foam pad (L 47 × W 39 × H 6 cm)	
	Week 7: Gaming on Foam pad (L 47 × W 39 × H 6 cm) plus 1 kg vest	
	Week 8: Gaming on Foam pad (L 47 × W 39 × H 6 cm) plus 1 kg vest	
	Week 9: Gaming on Foam pad (L 47 × W 39 × H 6 cm) plus 2 kg vest	
	Week 10: Gaming on Foam pad (L 47 × W 39 × H 6 cm) plus 2 kg vest	
Post-training assessment	Children were assessed on MABC-2, SR, balance, and agility tests	3 weeks

#### Measures Taken at Pre- and Post-Training

##### Shuttle Run Test

The Shuttle Run (SR) test was used to evaluate aerobic fitness performance. The test was performed in accordance with recommendations proposed by Leger and Lambert (1982). The highest level achieved by participants was recorded and used in the analysis. The SR test has been demonstrated to have good test-retest reliability (ICC = 0.93) and validity (*r* = 0.72) and had been used in an earlier study involving South African children (Ferguson et al., 2013).

##### Movement Assessment Battery for Children Test, Second Edition (MABC-2 Test)

The MABC-2 test measures motor performance in children aged 3–16 years (Hendersen et al., 2007). The test involves eight motor tasks divided into three categories: manual dexterity, aiming and catching, and balance skills. The raw scores for each category were converted to standard scores and percentiles, and these values were summed to generate overall percentile scores. Percentiles can be interpreted as follows: normal motor development (≥25th percentile), being at risk for motor difficulties (5th percentile < x ≤ 16th percentile), or having significant motor difficulty (≤ 5th percentile) (Hendersen et al., 2007). The MABC-2 test has demonstrated good validity and test-retest reliability with ICC values ranging from 0.92 to 0.98 (Hendersen et al., 2007).

##### Ladder Agility Test (LAT)

The LAT was used to measure children's agility performance (Smits-Engelsman et al., 2019). The LAT consists of two off-the-shelf 4 m agility ladders. These ladders were adapted by moving the 10 crossbars and fixating them at different distances to create two different ladders, “normal” and “accuracy.” For the “normal ladder” all the squares had equal sizes; the 10 3-cm broad yellow bars were separated by 36 cm. The “accuracy ladder” had unequal spaces between the bars to increase the spatial demands of the task. The first square was 44 cm in length, and the distance between the bars in successive squares decreased by 2 cm (44, 42, 40, 38, 36, 34, 32, 30, and 28 cm). Hence, both ladders had equal length (354 cm).

Both ladders with rungs of nine squares were positioned on the floor, and a designated turning point was marked 50 cm at one end of the ladders. From one end of the ladder, participants were instructed to step into each square as quickly as possible, make a 180° turn at the other end, and run back to the starting position. Participants began with both feet behind the crossbar of the first square and completed the test when they got back to the starting point. Upon hearing the “go signal,” the child was required to run forward using the required stepping pattern (single run: one foot in each square, or double run: two feet per square), turn at the turning point demarcated with a colored cone, and return to the starting point by following the same running pattern (Smits-Engelsman et al., 2019). Each participant performed two repetitions on each of the four items: type of ladder (normal and accuracy) with each running pattern (single or double run). Children had a 30 s rest between the two ladder types to ensure adequate recovery. The time taken to complete a full lap on each trial was recorded. Also, the number of mistakes produced during testing was recorded. A mistake was defined as either missing a square, placing the wrong foot or feet in the squares, and/or stepping on the bar separating two consecutive squares. The maximum number of mistakes allowed was three. If three or more mistakes in one run were made, an extra trial was given.

##### Foam Balance Test

Participants' balance was assessed using the foam balance test. For this test, a child was required to assume a one-legged stance on an Airex balance foam [i.e., a high-density closed-cell foam pad (47 × 39 × 6 cm, 0.7 kg)]. Participants' performance was timed using a stopwatch. Each subject completed two trials per leg in a maximum of 20 s. A 10-s rest was allowed between trials. A maximum of 5 s practice session was allowed prior to the start of each test so that subjects could gain some familiarity with the support surface. For all trials, the subjects placed their hands on their hips, and timing started when the opposite foot was lifted from the floor and stopped when the child could no longer control his or her posture or dropped the elevated foot. The best time recorded (in s) was used for the analysis.

##### 10 × 5 m Sprint Test-Straight

For this test, each participant was required to perform 10 quick runs over a distance of five m without stopping (Bonney et al., 2019). Colored cones were used to demarcate a 5-m running course in a designated hall. The participant begins at the starting point and runs toward the opposite end as quickly as possible. After every 5 m, the participant turns around and continues to run until 10 laps are completed. The time used to complete 10 laps was recorded (measured in s). This test was conducted individually under the supervision of a trained assessor. The test has good reliability in typically developing children (Bonney et al., 2019).

##### The Functional Strength Measure (FSM)

One upper (overarm throwing of a sandbag) extremity item and one lower extremity (standing-long-jump) item of the FSM (Aertssen et al., 2016) were used to assess functional muscle strength. These test items assess maximal explosive power (distance in cm). The FSM test has excellent psychometric properties in this population (Aertssen et al., 2016).

##### Körperkoordinationstest Für Kinder (KTK)

Two items of the Kiphard and Schilling (2007) were used to measure dynamic coordination and body control. The KTK consists of four test items: (a) walking backwards on a balance beam (3, 4.5, and 6 cm), (b) hopping over an obstacle, (c) jumping sideways over a wooden board, and (d) moving sideways using two wooden platforms. Specifically, two tests—jumping sideways, i.e., jumping from side to side, two-legged, for 15 s, and shifting platforms, i.e., moving sideways on two wooden boards for 20 s,—were used in this study. Two trials were given, and a summed score was used for the analysis. Test-retest reliability coefficients for the raw score are reported to be *r* = 0.95 (Kiphard and Schilling, 2007).

#### Measures Taken During the Training Period

##### Heart Rate

Heart rate was measured for each participant during the training with a Polar heart rate monitor (Polar S810). The device was strapped across the participant's chest, and heart rate readings were recorded with an accompanying wristwatch. Resting heart rate (HR) and peak heart rate were recorded, and the estimated maximum heart rate was computed using the formula: estimated maximum heart rate (EMHR_max_) = 206 – (0.88 × age) (Robergs and Landwehr, 2002). Also, the percentage of the estimated HR reached during the training was calculated to ascertain whether an individual child's maximum HR was above the recommended level.

##### Perceived Exertion

The Borg Rating of Perceived Exertion (RPE) scale was used to monitor the intensity of the exercises during the training (Borg, 1998). The Borg RPE scale consists of numerical values 6–20, where 6 means “no exertion at all” and 20 means “maximal exertion.” The Borg RPE scale is reported to be valid and reliable (Day et al., 2004).

##### Enjoyment Rating Scale

To quantify participants' enjoyment level during the training session, the Enjoyment scale was used (Jelsma et al., 2014). The Enjoyment scale contains five smiley faces with numeric scores (0–4, 0 meaning “Not fun at all,” and 4 “Super fun”). Participants were required to choose a smiley face to indicate their enjoyment level.

##### Training

The graded exergames training used in this study was designed using the Nintendo Wii games and was based on a published protocol (Bonney et al., 2018). In developing the intervention, two main criteria were used to select appropriate games: (1) games should require whole body movement to control the avatar, and (2) games should be responsive to external modifications without limiting playability. Based on these criteria, games such as the “Hula Hoop,” “Perfect 10,” “Jogging,” “Soccer Heading,” “Obstacle Course,” and “Torso Twists” were included. Foam pads (1.5 and 6 cm-thick closed cell foam pads) and vests with sandbags (with weights of 1 and 2 kg) were used to increase the physical demands of the selected games and to progressively increase the level of postural challenge required. A detailed description of the training is provided in Appendix 1 (Supplementary Material). Each participant was required to play 5–6 games twice for 30 min per session, twice weekly for 10 weeks. Half the children trained on Mondays and Wednesdays, and the other half on Tuesdays and Thursdays. Each 30-min training session started with warm-up games (e.g., “Basic Steps” or Jogging”) and included the performance of games from the three available game categories (anaerobic fitness, balance, and yoga). During the first 2 weeks, the participants were instructed to familiarize themselves with the selected Wii games; hence, no alterations were introduced throughout this period. From Week 3 to Week 10, games were adjusted to increase the postural demands and physiological load. This was done through the use of a foam pad and vest filled with two weights. The training was given to a maximum of six participants simultaneously in an enclosed room. Six Wii consoles and TVs were arranged and partitioned so that participants were not distracted by other players. Each session was supervised by one physical therapist and one fitness trainer. One person was responsible for fetching the children from class and fixing the heart rate monitors and weight vests. The other was solely responsible for supervising the training and ensuring that each child had enough time on task. If a child missed a training session, catch-up sessions were held on Fridays.

#### Data Analysis

Data were checked for normality and equality of variances, and appropriate parametric or non-parametric analyses were performed. Baseline differences in demographic characteristics, and motor performance between the training groups were calculated using chi-square tests (sex) or *t*-tests (age, BMI, MABC-2 total, and SR). To estimate the intensity of the training, averages of the RPE, and HR over 10 weeks were analyzed. The main effect of Time and the interaction of Time with Group on the HR, RPE, and Enjoyment scale were analyzed using repeated measures ANOVA with Time (10 weeks) as the within-group factor and Group (TD/DCD) as the between-group factor.

Changes in fitness and motor outcomes before and after training were analyzed using repeated measures ANOVA with time (pre-post) as within-group factors and group (TD/DCD) as the between-group factor. If interactions emerged, *post-hoc* tests were performed. The standardized mean difference was calculated by subtracting the mean of the scores at the post-test from the mean at the pretest and dividing this difference by the pooled standard deviation. The magnitude of the effect size was interpreted using Cohen's Conventions: small = 0.2, medium = 0.5, large = 0.8 (Cohen, 1988).

The individual peak HR was compared with the percentage of the estimated maximum HR needed for moderate intensity training. Next, the correlation between peak HR and RPE and between peak HR and Enjoyment scores was determined to ascertain whether greater exertion made playing the games less fun. To compensate for test-retest bias and determine change at an individual level, we calculated the number of children who improved more by estimating the measurement error (SEM) and the smallest detectable difference (SDD = 1.96 × SEM) on motor, sprint, and agility tests. All statistical analyses were performed using the Statistical Package for the Social Sciences (SPSS Inc., version 26), and the level of significance was set at *p* < 0.05.

## Results

### Group Differences Before Training

No group differences were found between DCD and TD groups with regard to age, gender, weight, height, or BMI. Significant differences were found between DCD and TD children on the MABC-2 and Shuttle Run tests (Table 2).

**Table 2:** Characteristics of TD and DCD groups at the start of the training.

**Variable**	**TD (*****n*** **=** **28)**		**DCD (*****n=*** **32)**	
	**Mean**	***SD***	**Mean**	***SD***
Age	9.82	1.42	9.31	1.12
BMI	16.87	2.94	17.46	3.81
Shuttle run level^*^	2.39	0.24	2.25	0.31
MABC-2 total standard score^**^	9.25	1.48	5.13	1.70

* *Significantly different between TD and DCD at p < 0.05*.

** *Significantly different between TD and DCD at p < 0.001*.

### Comparison of Motor and Fitness Performance Between DCD and TD Groups

#### Pre-Post-Comparison

The means for pre- and post-test outcomes with statistics (main effect of Time) are shown in Table 3. After training, balance performance was better (MABC-2 Balance and Foam task in left leg). Large differences were found on the anaerobic and aerobic items (10 × 5 m and SR) as well as on the Ladder Agility Tasks (Table 3). On only two variables was an interaction effect for Time by Group found; for the MABC-2 total score [*F*_(1, 55)_ = 13.19; *p* < 0.001] and for the MABC-2 balance sub score [*F*_(1, 55)_ = 26.83; *p* < 0.0001], indicating larger improvement in the children with DCD compared to TD on this measure (Table 4 shows pre- and post-test means for the TD and DCD groups, separately).

**Table 3:** Comparison of motor and fitness performance before and after training (mean, standard deviation, *F*-value for the main effect of Time, *p*-values, and degrees of freedom).

**Variables**	**Time**				**Statistics**		
	**Pre**		**Post**				
	**Mean**	***SD***	**Mean**	***SD***	***F***	***p*-value**	***df***
MABC-2 total score (SS)^$^	6.88	2.57	8.05	2.40	15.44	**0.0001**	57
MABC-2 manual dexterity, (SS)	7.98	2.95	8.09	2.31	0.34	0.61	57
MABC-2 Aiming Catching, (SS)	7.43	2.83	8.09	3.54	2.07	0.16	57
MABC-2 balance, (SS)^$^	7.96	3.19	9.57	2.84	14.80	**0.0001**	57
Foam left (s)	14.07	6.02	16.04	4.86	5.44	**0.024**	54
Foam right (s)	15.17	6.00	16.04	5.56	1.07	0.31	53
Agility ladder: Normal (s)	10.75	2.25	9.22	1.59	41.13	**0.0001**	53
Agility ladder: Accuracy (s)	11.07	2.33	9.42	1.61	43.96	**0.0001**	53
KTK platform (number)	34.26	6.84	39.81	7.35	27.80	**0.0001**	50
KTK side jumps (number)	49.30	12.82	55.20	14.37	8.09	**0.007**	40
10 × 5 m: sprint (s)	24.20	2.99	22.75	2.267	12.37	**0.001**	40
Long jump (cm)	124.60	34.40	124.70	29.73	0.73	0.38	30
Overhand throw (cm)	222.77	51.68	226.20	59.89	0.21	0.65	30
Shuttle run level	2.26	0.239	2.90	1.23	8.38	**0.008**	27

$ *Significant Time by Group interaction. Significant values are printed bold*.

**Table 4:** Means and standard deviations of motor and fitness performance variables before and after training for the TD and DCD groups for the children who have participated in the post-test.

**Variables**	**TD**					**DCD**				
	**Pre**		**Post**			**Pre**		**Post**		
	**Mean**	***SD***	**Mean**	***SD***	***n***	**Mean**	***SD***	**Mean**	***SD***	***n***
MABC-2 total score (SS),	9.12	1.5	9.16	2.1	25	5.13	1.7	7.16	2.2	32
MABC-2 manual dexterity, (SS)	9.40	2.8	9.08	2.2	25	6.78	2.5	7.25	2.2	32
MABC-2 aiming catching, (SS)	8.72	2.7	9.20	3.1	25	6.39	2.5	6.97	3.3	32
MABC-2 balance, (SS)	9.96	1.9	9.68	2.7	25	6.31	3.1	9.67	3.0	32
Foam left (s)	14.56	6.7	16.12	6.5	25	13.65	5.5	12.58	6.2	29
Foam right (s)	17.13	4.8	17.5	4.5	25	13.55	6.5	14.82	6.1	29
Agility ladder: Normal (s)	10.60	2.1	8.97	1.5	24	12.11	2.7	10.08	1.6	29
Agility ladder: Accuracy (s)	10.66	2.0	9.17	1.5	24	12.07	2.6	10.45	1.7	29
KTK platform (number)	37.70	5.4	41.87	5.9	23	30.62	6.7	36.19	7.8	27
KTK side jumps (number)	56.71	10.2	61.06	13.2	17	43.82	11.9	50.86	13.9	23
10 × 5m – sprint (s)	23.41	3.02	21.86	2.3	20	24.98	2.8	23.65	1.9	20
Long jump (cm)	133.67	28.29	128.07	30.6	15	115.53	38.4	121.33	29.5	15
Overhand throw (cm)	249.47	53.5	242.47	59.7	15	196.06	33.8	209.93	57.4	15
Shuttle run level	2.35	0.14	3.22	1.6	12	2.19	0.28	2.64	0.79	15

Table 5 shows that the training effect was rather specific; aerobic and anaerobic capacity and agility showed large effect sizes. Balance improved moderately, whereas skills not trained (aiming and catching and manual dexterity) and explosive power (FSM) showed no improvement.

**Table 5:** Effect sizes for the changes in motor and fitness performance variables.

**Variables**	**Cohen's *d***
Shuttle run level	0.87
Agility ladder: Accuracy (s)	−0.84
Agility ladder: Normal (s)	−0.80
KTK platform (number)	0.78
10 × 5 m sprint (s)	−0.55
MABC-2 balance (SS)	0.53
MABC-2 total score (ISS) (SS)	0.47
KTK side jumps (number)	0.43
Foam left (s)	0.36
MABC-2 aiming catching (SS)	Ns
Foam right (s)	Ns
MABC-2 manual dexterity (SS)	Ns
Long jump (cm)	Ns
Overhand throw (cm)	Ns

#### Individual Change

For some of the measures used in this study, SEM and SDD were known from psychometric studies (Holm et al., 2013; Bonney et al., 2019). In Table 6, the percentage of children who improved more than SEM and SDD is shown and indicates that at least half the children benefitted from the training more than the SDD on the test. Among children with DCD, 52% significantly improved on the LAT while this was 36% on the Balance sub score.

**Table 6:** Percentage of individuals with progress beyond the SEM and SDD per group.

**Variable**	**Total (%)**			**TD (%)**		**DCD (%)**	
	**≥SEM**	**≥SDD**	**Improvement**	**≥SEM**	**≥SDD**	**≥SEM**	**≥SDD**
Total MABC-2 (*n* = 57)	34	7	41	14	0	53	13
Cluster balance (*n* = 57)	19	23	42	7	4	27	36
Total agility ladder (*n* = 53)	28	40	68	50	25	10	52
10 × 5 sprint (*n* = 40)	28	23	51	20	30	35	15

#### Participants' Characteristics During the Training

**Figure 2:**
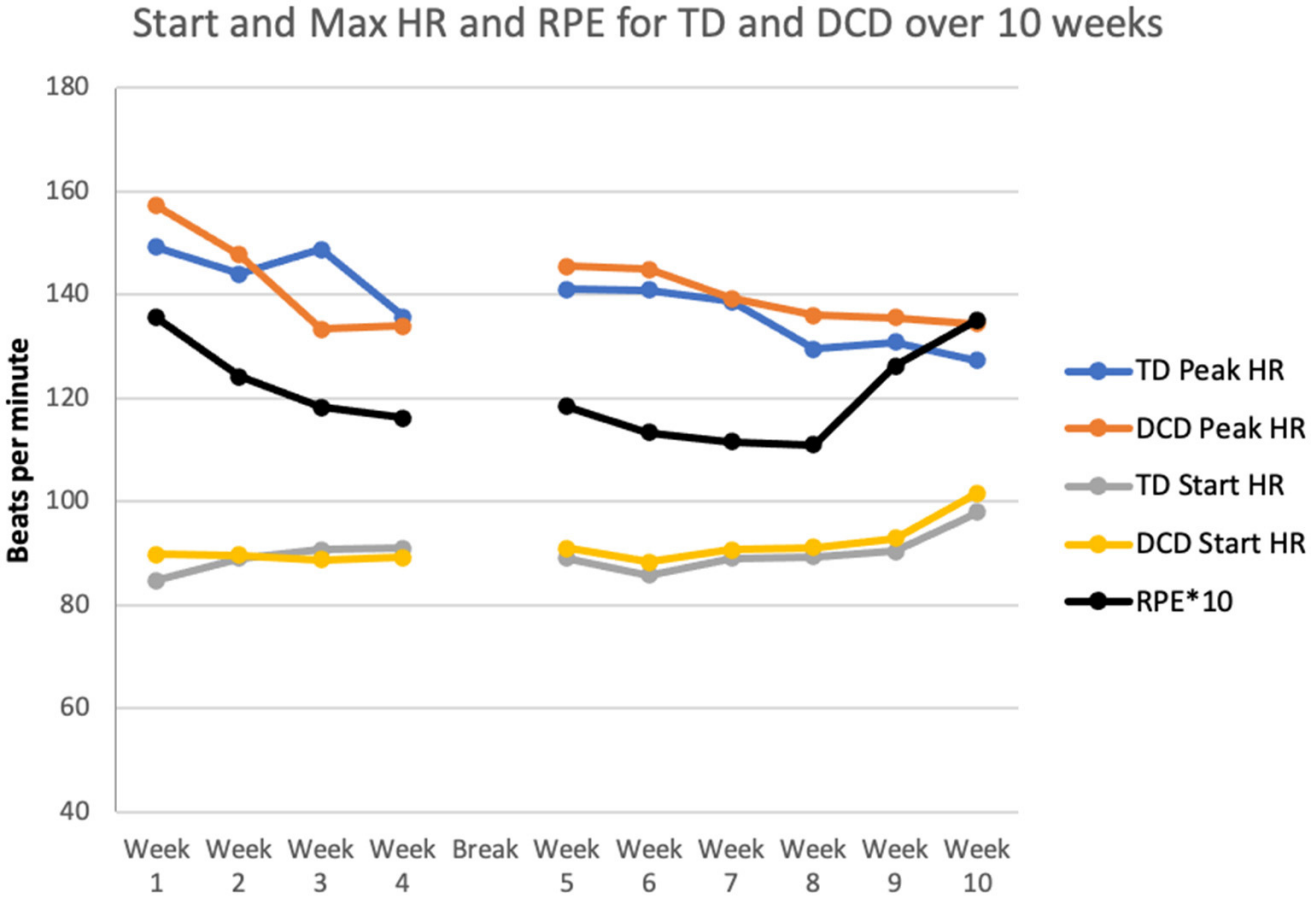
Comparison of heart rate (HR) and Rating of Perceived Exertion (RPE) scale for TD children and children with DCD during the 10 weeks of training.

The mean Max HR during the training was 139.3 ± 7.2 beats per minute (bpm) and was not different for the two groups [*F*_(1, 58)_ = 2.47; *p* = 0.12; TD 137.5 ± 7.2, DCD 140.26 ± 6.9]. The estimated max HR was 195.4 ± 1.1; 60% was 118.6 ± 0.67. In most of the sessions (7/10 of the weeks) the maximum heart rate measured reached at least 60% of the estimated max HR for all the children (Figure 2). Of all the HR readings, 30% were above the 70% level and 80% above the 60% estimated max HR level. This confirms that in most cases an adequate maximum level of intensity was reached. As shown in Figure 2, HR fluctuated over the 10 weeks [*F*_(9, 50)_ = 12.24; *p* < 0.001]. The changes reflect a gradual decrease in the first 4 weeks before the break and a small increase after the children returned to the study. Only four children did not reach max heart rate above 60% of estimated max HR in at least 7 of the 10 training weeks.

**Figure 3:**
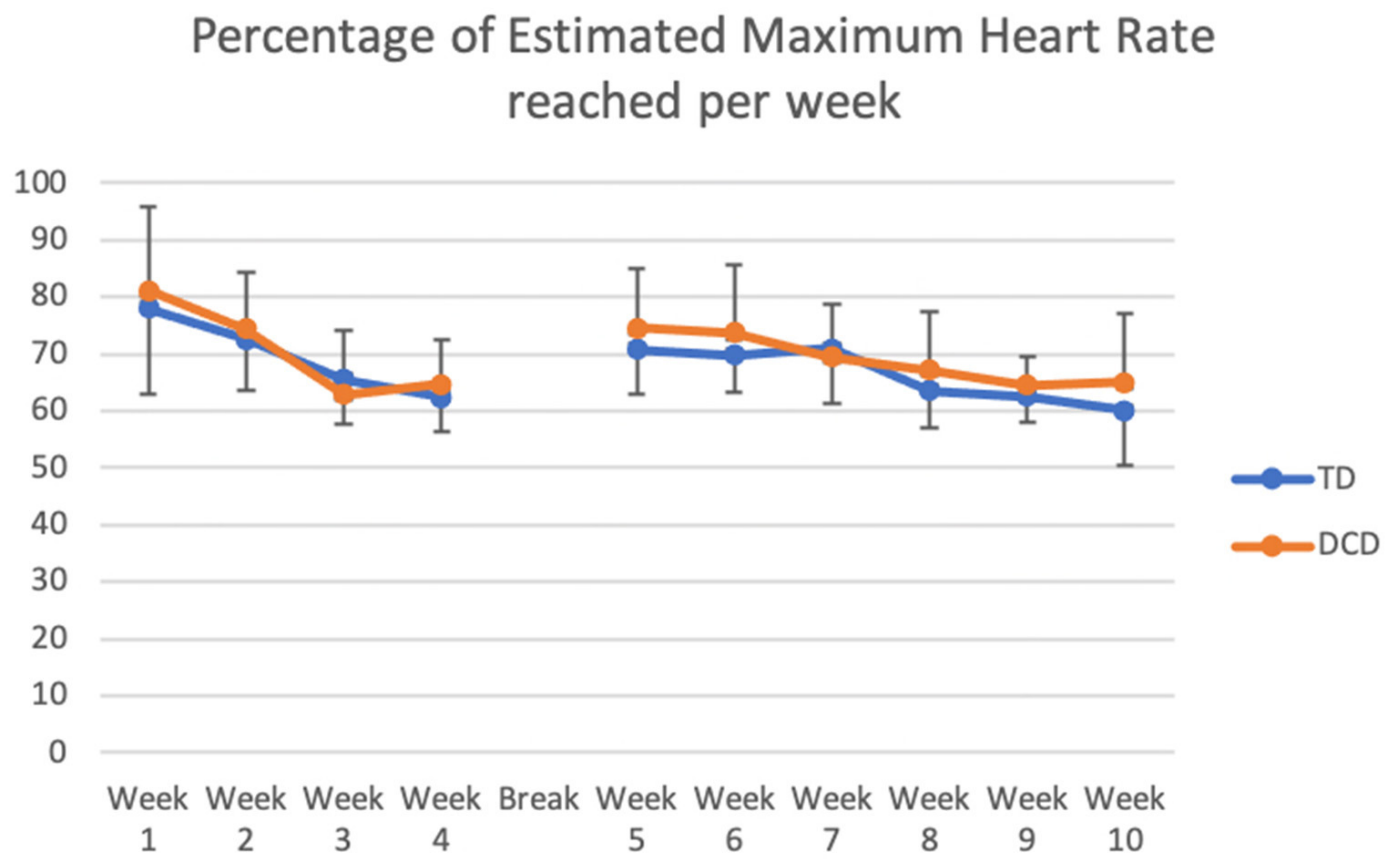
Comparison of the percentage of Estimated Maximum Heart Rate that TD children and children with DCD reached during the 10 weeks of training.

The mean perceived exertion was rated 12.1 (*SD* = 0.93) indicating “somewhat hard” exercise. The perception of the two groups regarding the intensity of the training was similar [*F*_(1, 58)_ = 0.008; *p* = 0.93)]. Variation over the 10 weeks is depicted in Figure 3 [*F*_(9, 50)_ = 13.27; *p* < 0.001]. The RPE went down gradually but increased for many children after the 2 kg vest for children was added.

**Figure 4:**
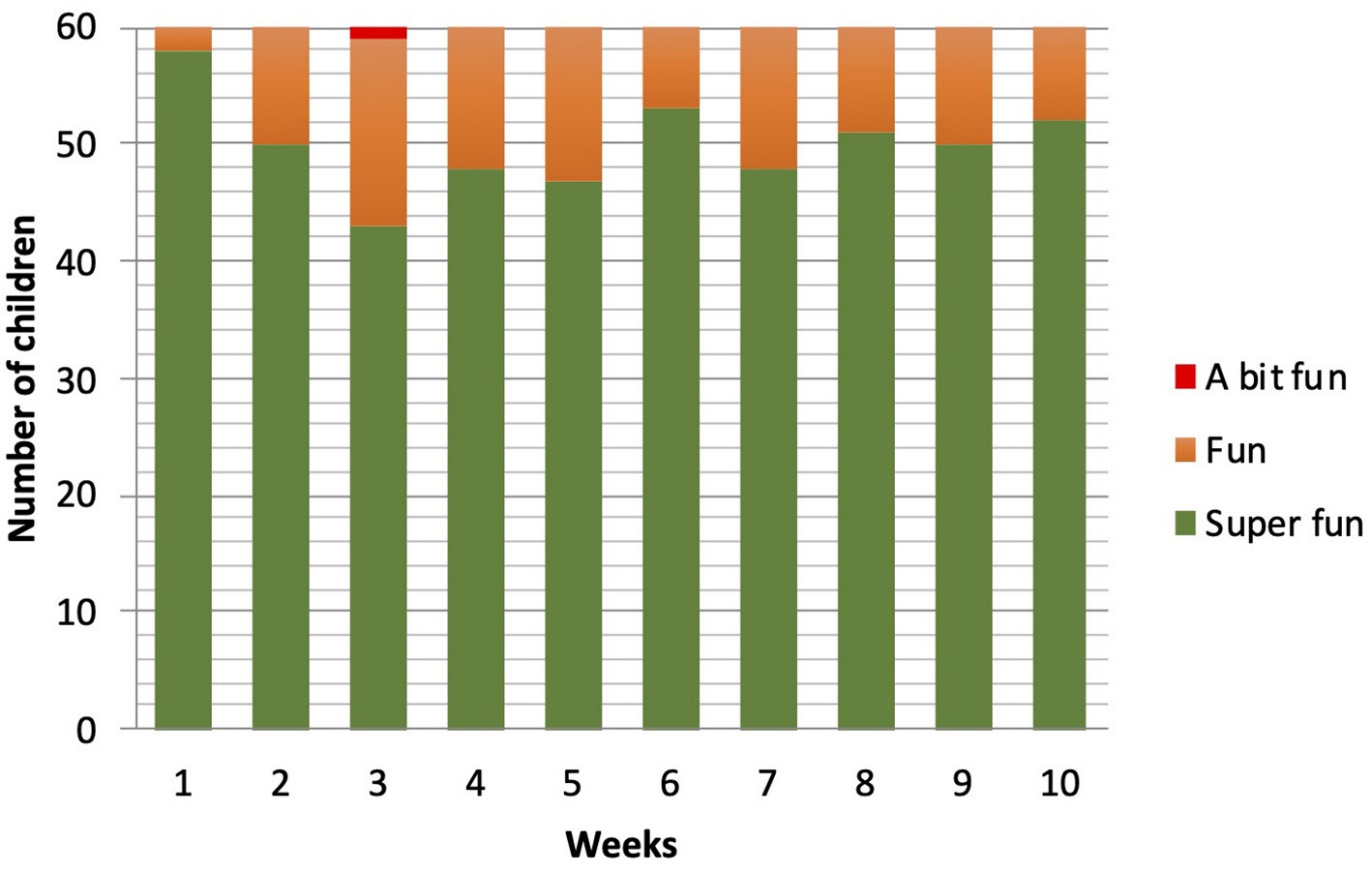
Enjoyment levels reported by the children during the 10 weeks of training.

The enjoyment in playing the games fluctuated between Super fun (score 4) and Fun (score 3). No differences between the ratings were found between the TD and DCD group [*F*_(1, 58)_ = 0.393; *p* = 0.53]. Only one child (DCD) in week 3 rated the training as “A bit fun.” “Boring” or “Not fun at all” was never scored. A main effect of time was found [*F*_(9, 50)_ = 3.63; *p* = 0.002]. More detailed analysis showed a cubic trend [*F*_(1, 58)_ = 4.43; *p* = 0.04]. There was a drop in enjoyment after week 1, the lowest value was found at training week 3, and then rating went up in the rest of the sessions. From week 7, the loads on the training were increased (week 7 and 8 with 1 kg extra; week 9 and 10 with 2 kg extra), and interestingly, this did not lead to a decrease in the number of children rating the training as “Super fun” (Figure 4).

A significant correlation (*r*_*p*_ = 0.47, *p* = 0.001) was found between the mean max HR during training and the mean RPE score the children gave after the training. No significant correlations were found between Enjoyment and mean RPE or Enjoyment and mean max HR. Children did not like the training less if it was perceived as harder or lighter.

## Discussion

This study was performed to examine the effects of graded exergames on motor and fitness performance in a sample of elementary school children with and without DCD. The main finding of this study was that children with DCD and TD children experienced gains in motor coordination (i.e., static and dynamic balance and total body coordination) and fitness performance (i.e., aerobic, anaerobic, and agility performance) after 10 weeks of training, which took place in a school setting. A significant interaction of Group by Time after training was observed and the DCD group was found to have larger improvements than the TD children in balance and total body coordination as measured on the MABC-2 test. Also, our study found that both groups of participants enjoyed the training and perceived its intensity to be high (an average of 12 points on the Borg scale). These findings provide new insights into the effects of graded exergames in elementary school children. Since this paper adapted and tested our earlier protocol in young children with and without DCD (Bonney et al., 2018), we can speculate that the current data seem to validate the graded exergames protocol in individuals with DCD.

There is very limited field research on skills training and fitness training in an elementary school setting. The current study provides additional data to physical education teachers, as well as pediatric, physical, and occupational therapists regarding the importance of graded exergames to enhance physical fitness in children with movement difficulties in school settings. However, the adoption of AVGs within the school environment will ultimately be determined by school staff and by practical constraints (e.g., availability and storage options for the equipment; Norris et al., 2016). The fact that we needed to break down our setup every afternoon and pack it into a secure storage room may be a real barrier for its use outside the experimental setting. Importantly, children with DCD in our study have no other options for intervention. Therefore, using AVGs during breaktimes could help them improve their skills and physical fitness, building confidence to take part in regular playground activities.

On this topic, there are currently limited data to which we can relate our findings. We found only one study that examined the feasibility of the graded Wii protocol in youth (females) with probable DCD (Bonney et al., 2018). In that study, it was reported that older school children demonstrated improvements in aerobic and anaerobic fitness after 14 weeks of training. Important points made for the children with DCD in that older age group may be even more relevant for this study. It could be that children with DCD felt more at ease without their peers watching them and the feedback provided by the games may have helped focus their attention inward instead of externally. Another benefit of this type of exercise is the ability to run the program in small spaces within the safety of the school premises. Last, the fact that exercising this way is a fun alternative to the traditional class-based exercise creates an opportunity to expose children with movement difficulties to a variety of options to develop a positive attitude toward exercise (Sheehan and Katz, 2013). This may help reverse withdrawal from physical activity, as motor competence and confidence in one's own motor skills are essential elements in the intrinsic desire to participate in physical activity (Higgs et al., 2008).

In the present investigation, both children with DCD and TD peers made significant gains in aerobic fitness, anaerobic fitness, agility performance, and balance (both static and dynamic) even though the training period was less than that of the previous study (10 vs. 14 weeks 30 vs. 60 min). Participants in our study reported high enjoyment and perceived exertion, which was similar in both groups. The collective experience of the children during the 10 weeks of training was positive (fun to awesome), and they reached the required 60% estimated peak HR. Remarkably, the effect sizes recorded for these gains after training were higher than what was reported in the previous study (Bonney et al., 2018). This is probably a consequence of the low start values, younger age (7–12 vs. 13–16 years) with lower BMI (17.1 vs. 27.5 kg/m^2^), and high enjoyment. It is also possible that due to the lack of organized physical activity in the school and neighboring communities, our 2 × 30 min fitness-enhancing graded program was adequate to stimulate short-term gains (after 10 weeks) in motor skills and fitness in these children.

A strength of our study rests on the fact that this study was conducted among 7- to 12-year-old children with DCD and age matched TD peers and adherence with our training was 100%. On the other hand, there are limitations. Our design had no control group, which makes it vulnerable to threats to internal validity. There is the possibility that other events (e.g., history, maturation, testing effects, and statistical regression) than the intervention administered might have caused the observed changes. Unfortunately, it was not possible to include a no-treatment control group because of ethical concerns associated with such a group. Furthermore, our results do not provide insight into the long-term effects of graded exergames on fitness performance in children with DCD. Despite these shortfalls and partly missing data, our study demonstrated the potential value of gradually grading exercises in children with DCD and provides data that serve to validate previous findings on the fitness-promoting benefits of graded exergames for individuals with DCD.

## Data Availability Statement

The raw data supporting the conclusions of this article will be made available by the authors, without undue reservation.

## Ethics Statement

The studies involving human participants were reviewed and approved by the study was approved by the Human Research Ethics Committee (HREC) of the University of Cape Town, South Africa (HREC: 209/2018). Written informed consent to participate in this study was provided by the participants' legal guardian/next of kin.

## Author Contributions

BS-E, EB, and GF: conceptualization, design, funding, and data acquisition. BS-E and EB: data analysis and interpretation as well as manuscript preparation. All authors critically reviewed and approved the final manuscript.

## Conflict of Interest

The authors declare that the research was conducted in the absence of any commercial or financial relationships that could be construed as a potential conflict of interest.

## References

[B1] Aertssen W. F. Ferguson G. D. Smits-Engelsman B. C. (2016). Reliability and structural and construct validity of the functional strength measurement in children aged 4 to 10 years. Phys. Ther. 96, 888–897. 10.2522/ptj.20140018 26586864

[B2] American Psychiatric Association (2013). Diagnostic and Statistical Manual of Mental Disorders, 5th Edn. Washington, DC: American Psychiatric Association.

[B3] Biddle S. J. Gorely T. Stensel D. J. (2004). Health-enhancing physical activity and sedentary behavior in children and adolescents. J. Sports Sci. 22, 679–701. 10.1080/02640410410001712412 15370482

[B4] Blank R. Barnett A. L. Cairney J. Green D. Kirby A. Polatajko H.. (2019). International clinical practice recommendations on the definition, diagnosis, assessment, intervention, and psychosocial aspects of developmental coordination disorder. Dev. Med. Child Neurol. 61, 242–285. 10.1111/dmcn.14132 30671947 PMC6850610

[B5] Bonney E. Aertssen W. Smits-Engelsman B. (2019). Psychometric properties of field-based anaerobic capacity tests in children with Developmental Coordination Disorder. Disabil. Rehabil. 41, 1803–1814. 10.1080/09638288.2018.1446189 29509037

[B6] Bonney E. Ferguson G. Smits-Engelsman B. (2017a). The efficacy of two activity-based interventions in adolescents with developmental coordination disorder. Res. Dev. Disabil. 71, 223–236. 10.1016/j.ridd.2017.10.013 29055242

[B7] Bonney E. Jelsma D. Ferguson G. Smits-Engelsman B. (2017b). Variable training does not lead to better motor learning compared to repetitive training in children with and without DCD when exposed to active video games. Res. Dev. Disabil. 62, 124–136. 10.1016/j.ridd.2017.01.013 28157565

[B8] Bonney E. Rameckers E. Ferguson G. Smits-Engelsman B. (2018). “Not just another Wii training”: a graded Wii protocol to increase physical fitness in adolescent girls with probable developmental coordination disorder-a pilot study. BMC Pediatr. 18:78. 10.1186/s12887-018-1029-7 29471799 PMC5822519

[B9] Boreham C. O. Twisk J. O. Murray L. I. Savage M. A. Strain J. J. Crain G. (2001). Fitness, fatness, and coronary heart disease risk in adolescents: the Northern Ireland Young Hearts Project. Med. Sci. Sports Exerc. 33, 270–274. 10.1097/00005768-200102000-00016 11224817

[B10] Borg G. (1998). Borg's Perceived Exertion and Pain Scales. Champaign, IL: Human kinetics.

[B11] Cairney J. Veldhuizen S. King-Dowling S. Faught B. E. Hay J. (2017). Tracking cardiorespiratory fitness and physical activity in children with and without motor coordination problems. J. Sci. Med. Sport 20, 380–385. 10.1016/j.jsams.2016.08.025 27760715

[B12] Castelli D. M. Hillman C. H. Buck S. M. Erwin H. E. (2007). Physical fitness and academic achievement in third-and fifth-grade students. J. Sport Exerc. Psychol. 29, 239–252. 10.1123/jsep.29.2.239 17568069

[B13] Cohen J. (1988). Statistical Power Analysis for the Behavioral Sciences. Lawrence Erlbaum Associates.

[B14] Corbin C. Pangrazi R. Franks B. (2000). Definitions: health, fitness, and physical activity, in President's Council on Physical Fitness and Sports. President's Council on Physical Fitness and Sports Research Digest series 3 (Silver Spring, MD: National Fitness Foundation), 1–9.

[B15] Day M. L. McGuigan M. R. Brice G. Foster C. (2004). Monitoring exercise intensity during resistance training using the session RPE scale. J. Strength Cond. Res. 18, 353–358. 10.1519/00124278-200405000-00027 15142026

[B16] Deuster P.A. (ed.). (1997). Navy Seal Physical Fitness Guide. Washington, DC: DIANE Publishing.

[B17] Faigenbaum A. D. Farrell A. Fabiano M. Radler T. Naclerio F. Ratamess N. A.. (2011). Effects of integrative neuromuscular training on fitness performance in children. Pediatr. Exerc. Sci. 23, 573–584. 10.1123/pes.23.4.573 22109781

[B18] Farhat F. Hsairi I. Baiti H. Cairney J. Mchirgui R. Masmoudi K.. (2015). Assessment of physical fitness and exercise tolerance in children with developmental coordination disorder. Res. Dev. Disabil. 45:210–219. 10.1016/j.ridd.2015.07.023 26263407

[B19] Faught B. E. Hay J. A. Cairney J. Flouris A. (2005). Increased risk for coronary vascular disease in children with developmental coordination disorder. J. Adolesc. Health 37, 376–380. 10.1016/j.jadohealth.2004.09.021 16227122

[B20] Faul F. Erdfelder E. Lang A. G. Buchner A. (2007). G^*^Power 3: a flexible statistical power analysis program for the social, behavioral, and biomedical sciences. Behav. Res. Methods 39, 175–191. 10.3758/BF03193146 17695343

[B21] Ferguson G. D. Aertssen W. F. Rameckers E. A. Jelsma J. Smits-Engelsman B. C. (2014). Physical fitness in children with developmental coordination disorder: measurement matters. Res. Dev. Disabil. 35, 1087–1097. 10.1016/j.ridd.2014.01.031 24582141

[B22] Ferguson G. D. Jelsma D. Jelsma J. Smits-Engelsman B. C. (2013). The efficacy of two task-orientated interventions for children with Developmental Coordination Disorder: Neuromotor Task Training and Nintendo Wii Fit training. Res. Dev. Disabil. 34, 2449–2461. 10.1016/j.ridd.2013.05.007 23747936

[B23] Hammond J. Jones V. Hill E. L. Green D. Male I. (2014). An investigation of the impact of regular use of the W ii F it to improve motor and psychosocial outcomes in children with movement difficulties: a pilot study. Child Care Health Dev. 40, 165–175. 10.1111/cch.12029 23363371

[B24] Hay J. Missiuna C. (1998). Motor proficiency in children reporting low levels of participation in physical activity. Can. J. Occup. Ther. 65, 64–71. 10.1177/000841749806500203

[B25] Henderson S. E. Sugden D. A. Barnett A. L. (2007). Movement Assessment Battery for Children-2: Examiner's Manual, 2nd Edn. London: Pearson Assessment.

[B26] Higgs C. Balyi I. Way R. Cardinal C. Norris S. Bluechardt M. (2008). Developing Physical Literacy: A Guide for Parents of Children Ages 0 to 12. Vancouver, BC: Canadian Sport Centres.

[B27] Holm I. Tveter A. T. Aulie V. S. Stuge B. (2013). High intra- and inter-rater chance variation of the movement assessment battery for children 2, ageband 2. Res. Dev. Disabil. 34, 795–800. 10.1016/j.ridd.2012.11.002 23220056

[B28] Janssen I. LeBlanc A. G. (2010). Systematic review of the health benefits of physical activity and fitness in school-aged children and youth. Int. J. Bev. Nutr. 7:40. 10.1186/1479-5868-7-40 20459784 PMC2885312

[B29] Jelsma D. Geuze R. H. Mombarg R. Smits-Engelsman B. C. (2014). The impact of Wii fit intervention on dynamic balance control in children with probable developmental coordination disorder and balance problems. Hum. Mov. Sci. 33, 404–418. 10.1016/j.humov.2013.12.007 24444657

[B30] Kiphard E.J. Schilling F. (2007). Körperkoordinationtest für Kinder, 2nd Edn. Weinheim: Beltz Test. 5511840

[B31] Kolimechkov S. Petrov L. Alexandrova A. (2019). Alpha-fit test battery norms for children and adolescents from 5 to 18 years of age obtained by a linear interpolation of existing European physical fitness references. Eur. J. Phys. Educ. Sport Sci. 5, 65–78. 10.5281/zenodo.2546360

[B32] Lang J. J. Tomkinson G. R. Janssen I. Ruiz J. R. Ortega F. B. Léger L.. (2018). Making a case for cardiorespiratory fitness surveillance among children and youth. Exerc. Sport Sci. Rev. 46, 66–75. 10.1249/JES.0000000000000138 29346159

[B33] Leger L. A. Lambert J. (1982). A maximal multistage 20-m shuttle run test to predict VO_2_ max. Eur. J. Appl. Physiol. 49, 1–2. 10.1007/BF00428958 7201922

[B34] Lifshitz N. Raz-Silbiger S. Weintraub N. Steinhart S. Cermak S. A. Katz N. (2014). Physical fitness and overweight in Israeli children with and without developmental coordination disorder: gender differences. Res. Dev. Disabil. 35, 2773–2780. 10.1016/j.ridd.2014.07.020 25086737

[B35] Mentiplay B. F. FitzGerald T. L. Clark R. A. Bower K. J. Denehy L. Spittle A. J. (2019). Do video game interventions improve motor outcomes in children with developmental coordination disorder? A systematic review using the ICF framework. BMC Pediatr. 19:22. 10.1186/s12887-018-1381-7 30651097 PMC6335818

[B36] Norris E. Hamer M. Stamatakis E. (2016). Active video games in schools and effects on physical activity and health: a systematic review. J. Pediatr. 172, 40–46.e5. 10.1016/j.jpeds.2016.02.001 26947570

[B37] Ortega F. B. Ruiz J. R. Castillo M. J. Sjöström M. (2008). Physical fitness in childhood and adolescence: a powerful marker of health. Int. J. Obes. 32, 1–11. 10.1038/sj.ijo.0803774 18043605

[B38] Rivilis I. Hay J. Cairney J. Klentrou P. Liu J. Faught B. E. (2011). Physical activity and fitness in children with developmental coordination disorder: a systematic review. Res. Dev. Disabil. 32, 894–910. 10.1016/j.ridd.2011.01.017 21310588

[B39] Robergs R. A. Landwehr R. (2002). The surprising history of the “HRmax = 220-age” equation. J. Exerc. Physiol. Online 5, 1–10.

[B40] Sheehan D. P. Katz L. (2013). The effects of a daily, 6-week exergaming curriculum on balance in fourth grade children. J. Sport Health Sci. 2, 131–137. 10.1016/j.jshs.2013.02.002

[B41] Smits-Engelsman B. Aertssen W. Bonney E. (2019). Reliability and validity of the ladder agility test among children. Pediatr. Exerc. Sci. 31, 370–378. 10.1123/pes.2018-0117 30786827

[B42] Smits-Engelsman B. Vincon S. Blank R. Quadrado V. H. Polatajko H. Wilson P. H. (2018). Evaluating the evidence for motor-based interventions in developmental coordination disorder: a systematic review and meta-analysis. Res. Dev. Disabil. 74, 72–102. 10.1016/j.ridd.2018.01.002 29413431

[B43] Tomkinson G. R. Lang J. J. Tremblay M. S. (2019). Temporal trends in the cardiorespiratory fitness of children and adolescents representing 19 high-income and upper middle-income countries between 1981 and 2014. Br. J. Sports Med. 53, 478–486. 10.1136/bjsports-2017-097982 29084727

[B44] Tsiotra G. D. Nevill A. M. Lane A. M. Koutedakis Y. (2009). Physical fitness and developmental coordination disorder in Greek children. Pediatr. Exerc. Sci. 21, 186–195. 10.1123/pes.21.2.186 19556624

[B45] Wittberg R. A. Northrup K. L. Cottrell L. A. (2012). Children's aerobic fitness and academic achievement: a longitudinal examination of students during their fifth and seventh grade years. Am. J. Public Health 102, 2303–2307. 10.2105/AJPH.2011.300515 22698045 PMC3519293

