# Supplementary material for: Effects of Graded Exergames on Fitness Performance in Elementary School Children With Developmental Coordination Disorder
Source: Front Sports Act Living. 2021 Apr 22;3:653851. doi: 10.3389/fspor.2021.653851 (PMC8100245; doi:10.3389/fspor.2021.653851)
Supplement: Supplementary file 1 [file Table_1.DOCX]

**The Graded Wii Protocol for children between 7-12 (adapted from Bonney et al., 2008)**

1. Heart rate is measured before, during and after training using POLARS
2. After training exertion and enjoyment was measured using BORG RPE SCALE and ENJOYMENT SCALE

| **Weeks#** | **Training #** | **List of Games** | **Number of reps per session** | **Level of Adaptation to be made** |
| --- | --- | --- | --- | --- |
| 1  **30 mins session** | T1 | 1. **Jogging (short distance)** 2. **Hula Hoop** 3. **Lunge** 4. **Half Moon** 5. **Soccer heading** | 2  2  2  2  2 | None |
|  | T2 | 1. **Jogging (Short distance)** 2. **Penguin slide** 3. **Single leg extension** 4. **Rowing squat** 5. **Warrior** 6. **Obstacle course** | 2  2  2  2  2  2 | None |
| 2  **30 mins session** | T3 | 1. **Jogging (Short distance)** 2. **Table tilt** 3. **Perfect 10** 4. **Jack Knife** 5. **Sun salutation** | 2  2  2  2  2 | None |
|  | T4 | 1. **Jogging (Short distance)** 2. **Boxing** 3. **Penguin slide** 4. **Soccer heading** 5. **Lunge** 6. **Tree** | 2  2  2  2  2  2 | None |
| 3  **30 mins session** | T5 | 1. **Basic steps** 2. **Torso twists** 3. **Perfect 10** 4. **Single leg extension** 5. **Half Moon** | 2  2  2  2  2 | Airex pads (2”) on balance board to increase balance |
|  | T6 | 1. **Jogging (Long distance)** 2. **Obstacle course** 3. **Rowing squat** 4. **Single leg twist** 5. **Penguin slide** 6. **Warrior** | 2  2  2  2  2  2 | Airex pads (2”) on balance board to increase balance |
| **30 mins session** | T7 | 1. **Basic steps** 2. **Table tilt** 3. **Soccer heading** 4. **Single leg extension** 5. **Half Moon** | 2  2  2  2  2 | Airex pads (2”) on balance board to increase balance |
|  | T8 | 1. **Jogging (Long distance)** 2. **Obstacle course** 3. **Penguin slide** 4. **Rowing squat** 5. **Single leg twist** 6. **Palm tree** | 2  2  2  2  2  2 | Airex pads (2”) on balance board to increase balance |
| 5  **30 mins session** | T9 | 1. **Jogging (Long distance)** 2. **Hula Hoop** 3. **Soccer heading** 4. **Lunge** 5. **Half Moon** | 2  2  2  2  2 | Foam pads (4”) on balance board to increase balance |
|  | T10 | 1. **Jogging (Long distance)** 2. **Penguin slide** 3. **Obstacle course** 4. **Single leg extension** 5. **Rowing squat** 6. **Warrior** | 2  2  2  2  2  2 | Foam pads (4”) on balance board to increase balance |
| 6  **30 mins session** | T11 | 1. **Jogging (Long distance)** 2. **Table tilt** 3. **Perfect 10** 4. **Jack Knife** 5. **Sun salutation** | 2  2  2  2  2 | Foam pads (4”) on balance board to increase balance |
|  | T12 | 1. **Jogging (Long distance)** 2. **Boxing** 3. **Penguin slide** 4. **Soccer heading** 5. **Lunge** 6. **Tree** | 2  2  2  2  2  2 | Foam pads (4”) on balance board to increase balance |
| 7  **30 mins session** | T13 | 1. **Jogging (Long distance)** 2. **Hula Hoop** 3. **Table tilt** 4. **Torso twist** 5. **Warrior** | 2  2  2  2  2 | Foam pads (4”) on balance board to increase balance  Wear exercise Vest with **1kg** weight |
|  | T14 | 1. **Jogging (Long distance)** 2. **Basic steps** 3. **Soccer heading** 4. **Rhythm boxing** 5. **Rowing squat** 6. **Half Moon** | 3  2  2  2  2  2 | Foam pads (4”) on balance board to increase balance  Wear exercise Vest with **1kg** weight |
| 8  **30 mins session** | T15 | 1. **Basic steps** 2. **Penguin slides** 3. **Soccer heading** 4. **Lunge** 5. **Palm tree** | 2  2  2  2  2 | Foam pads (4”) on balance board to increase balance  Wear exercise Vest with **1kg** weight |
|  | T16 | 1. **Jogging (Long distance)** 2. **Obstacle course** 3. **Penguin slide** 4. **Rowing squat** 5. **Single leg twist** 6. **Half Moon** | 2  2  2  2  2  2 | Foam pads (4”) on balance board to increase balance  Wear exercise Vest with 1**kg** weight |
| 9  **30 mins session** | T17 | 1. **Basic steps** 2. **Table tilt** 3. **Soccer heading** 4. **Single leg extension** 5. **Tree** | 2  2  2  2  2 | Foam pads (4”) on balance board to increase balance  Wear exercise Vest with **2kg** weight |
|  | T18 | 1. **Basic steps** 2. **Jogging (Long distance)** 3. **Table tilt** 4. **Perfect 10** 5. **Jack Knife** 6. **Sun salutation** | 2  2  2  2  2  2 | Foam pads (4”) on balance board to increase balance  Wear exercise Vest with **2kg** weight |
| 10  **30 mins session** | T19 | 1. **Jogging (Long distance)** 2. **Hula Hoop** 3. **Table tilt** 4. **Torso twist** 5. **Warrior** | 2  2  2  2  2 | Foam pads (4”) on balance board to increase balance  Wear exercise Vest with **2kg** weight |
|  | T20 | 1. **Jogging (Long distance)** 2. **Basic steps** 3. **Soccer heading** 4. **Rhythm boxing** 5. **Rowing squat** 6. **Half Moon** | 2  2  2  2  2  2 | Foam pads (4”) on balance board to increase balance  Wear exercise Vest with **2kg** weight |
